# A Reconstruction Algorithm for Temporally Aliased Seismic Signals Recorded by the InSight Mars Lander

**DOI:** 10.1029/2020EA001234

**Published:** 2021-08-11

**Authors:** David Sollberger, Cedric Schmelzbach, Fredrik Andersson, Johan O. A. Robertsson, Nienke Brinkman, Sharon Kedar, William B. Banerdt, John Clinton, Martin van Driel, Raphael Garcia, Domenico Giardini, Matthias Grott, Thomas Haag, Troy L. Hudson, Philippe Lognonné, Jan ten Pierick, William Pike, Tilman Spohn, Simon C. Stähler, Peter Zweifel

**Affiliations:** ^1^ Institute of Geophysics ETH Zürich Zürich Switzerland; ^2^ Jet Propulsion Laboratory California Institute of Technology Pasadena CA USA; ^3^ Institut Supérieur de l'Aéronautique et de l'Espace SUPAERO Toulouse France; ^4^ DLR Institute of Planetary Research Berlin Germany; ^5^ Université de Paris Institut de Physique du globe de Paris CNRS Paris France; ^6^ Department of Electrical and Electronic Engineering Imperial College London South Kensington Campus London UK

**Keywords:** Mars, seismic exploration, aliasing, signal processing, signal reconstruction

## Abstract

In December 2018, the NASA InSight lander successfully placed a seismometer on the surface of Mars. Alongside, a hammering device was deployed at the landing site that penetrated into the ground to attempt the first measurements of the planetary heat flow of Mars. The hammering of the heat probe generated repeated seismic signals that were registered by the seismometer and can potentially be used to image the shallow subsurface just below the lander. However, the broad frequency content of the seismic signals generated by the hammering extends beyond the Nyquist frequency governed by the seismometer's sampling rate of 100 samples per second. Here, we propose an algorithm to reconstruct the seismic signals beyond the classical sampling limits. We exploit the structure in the data due to thousands of repeated, only gradually varying hammering signals as the heat probe slowly penetrates into the ground. In addition, we make use of the fact that repeated hammering signals are sub‐sampled differently due to the unsynchronized timing between the hammer strikes and the seismometer recordings. This allows us to reconstruct signals beyond the classical Nyquist frequency limit by enforcing a sparsity constraint on the signal in a modified Radon transform domain. In addition, the proposed method reduces uncorrelated noise in the recorded data. Using both synthetic data and actual data recorded on Mars, we show how the proposed algorithm can be used to reconstruct the high‐frequency hammering signal at very high resolution.

## Key Points

- Hammering of the InSight heat probe generates high‐frequency seismic signals that exceed the Nyquist frequency of the seismometer
- We developed a new data acquisition and reconstruction workflow that allows for the recovery of the full‐bandwidth hammering signals
- During hammering, we deliberately turned off the seismometer's anti‐aliasing filters and reconstructed the aliased signal using a sparseness‐promoting algorithm

## 1 Introduction

The NASA InSight mission successfully landed on Mars in November 2018 (Banerdt et al., 2020). Since then, the SEIS package, consisting of two three‐component seismometers (Lognonné et al., 2019), and the heat flow and physical properties package (${HP}^{3}$) (Spohn et al., 2018) were deployed directly onto the surface of Mars. ${HP}^{3}$ consists of a self‐hammering probe, referred to as the “mole,” that penetrates into the shallow subsurface of the Martian regolith with the aim to take thermal conductivity and temperature measurements in order to better understand the Martian planetary heat flow. The hammering mechanism of the mole is designed to slowly dig into the regolith at a rate of about 0.1–1 mm per hammer stroke (Kedar et al., 2017). This means that thousands of repeated hammer strokes are needed to reach the target depth of 5 m.

${HP}^{3}$ hammering generates seismic signals that are recorded by SEIS. These signals can potentially be used to image the shallow subsurface just below the lander (Golombek et al., 2018; Kedar et al., 2017). However, the seismic analysis of the ${HP}^{3}$ hammering signals does not address one of the primary mission goals and the experiment was not conceived before finalizing the system design. Therefore, the data acquisition for this opportunistic experiment had to be implemented with the constraints given by the already designed seismic data acquisition flow. Hence, the need to develop the reconstruction workflow discussed in this paper.

**Figure 1:**
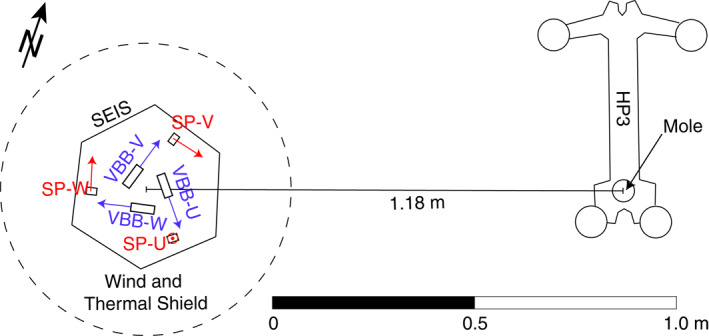
Configuration of SEIS and ${HP}^{3}$ on Mars. The orientation and location of the three components of the short‐period (SP) and very broadband (VBB) seismometers are marked in red and blue, respectively. The azimuths of the SP‐U, SP‐V, and SP‐W axes from North are 285°, 105.2°, and 345.3°, with an inclination from the horizontal of −89.9°, 0°, and 0°, respectively. The axes of the VBB sensor are all inclined with respect to the horizontal providing a set of three close‐to‐orthogonal components in a so‐called Galperin configuration. See Lognonné et al. (2019) for details.

SEIS is deployed in close proximity to the ${HP}^{3}$ mole at a distance of 1.18 m (Figure 1). As a result, the travel times of seismic waves generated by the hammering of the mole are extremely short (just a few milliseconds). In order to extract subsurface information from the seismic data (such as seismic velocity and reflectivity), it is thus of crucial importance to have a high temporal resolution for both the recorded seismic signal and the origin time of each mole stroke (i.e., the time the hammer stroke occurs). The latter is known with an accuracy of 1.7 milliseconds from the measurements of an accelerometer that is mounted inside the mole (Spohn et al., 2018). In this paper, we develop a method to additionally increase the temporal resolution of the recorded seismograms beyond the nominal sampling rate of the seismometer. Increasing the temporal resolution is a critical step since the nominal sampling interval of SEIS is longer than the expected seismic travel time between ${HP}^{3}$ and SEIS, effectively preventing the extraction of seismic velocities (Kedar et al., 2017).

**Figure 2:**
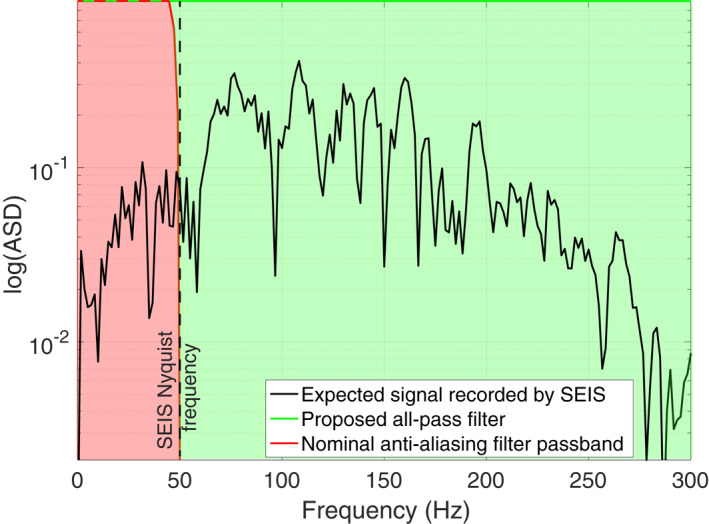
Amplitude spectral density of an ${HP}^{3}$ hammering seismic signal obtained in an analog experiment on Earth. The response of the nominal SEIS anti‐aliasing filter is shown in red. The proposed digital all‐pass filter passes information throughout the complete bandwidth (green). As a result, the recorded seismic signals will be aliased by several factors when downsampled to 100 samples per second.

SEIS is operated with on‐board digital anti‐aliasing filters to prepare the seismic information to be returned to Earth with a maximum sampling rate of 100 samples per second (sps). This sampling rate provides sufficient temporal resolution for most of the anticipated Martian seismic signals such as marsquakes and meteorite impacts (Giardini et al., 2020; Lognonné et al., 2019). However, the impulsive seismic signals generated by ${HP}^{3}$ hammering are very broad‐band and may contain frequencies up to and beyond 250 Hz (Kedar et al., 2017). The application of the nominal anti‐aliasing filter would thus result in a severe loss of information during acquisition. Figure 2 shows the signal of a single hammer stroke measured using a commercial seismometer in an analog experiment conducted on Earth in the Nevada desert. The pass region of the nominal SEIS anti‐aliasing filter is marked in red. Note how a significant portion of the information including the dominant signal energy between 100 and 150 Hz would be lost using the nominal anti‐aliasing filter.

Given the fact that the idea of using ${HP}^{3}$ as a seismic source was conceived after the implementation of the seismic acquisition hardware, the InSight science team had to find ways to circumvent limitations of the existing acquisition hardware, such as the insufficient sampling rate. With the goal to enable the analysis of seismic information beyond the highest nominal Nyquist frequency of SEIS (i.e., 50 Hz), we designed a data acquisition and reconstruction workflow that consists of (a) recording aliased data by replacing the nominal anti‐aliasing finite‐impulse response (FIR) filters by all‐pass filters and (b) reconstructing the data at a high sampling rate using a sparseness‐promoting algorithm. We illustrate the success of our method in recovering the high frequency information from the hammering signals using both synthetic data and actual data from Mars.

The ${HP}^{3}$–SEIS experiment marks, to the best of our knowledge, the first active seismic experiment ever conducted on Mars (Brinkman et al., 2019). A similar robotic active seismic experiment on an extraterrestrial object has only been attempted once before on the comet 67P Churyumov–Gerasimenko during the Rosetta mission and allowed for the extraction of the comet's elastic properties (Knapmeyer et al., 2018). On the lunar surface, the Apollo astronauts conducted active seismic profiling experiments using mortar and explosive sources (Brzostowski & Brzostowski, 2009; Nunn et al., 2020), in order to characterize the shallow subsurface structure at the Apollo 14, 16, and 17 landing sites (Cooper et al., 1974). In recent years, the Apollo active seismic data have been re‐processed with modern analysis tools that allowed for the extraction of novel information on the near‐surface structure of the Moon (Haase et al., 2019; Heffels et al., 2017; Larose et al., 2005; Phillips & Weber, 2020; Sens‐Schönfelder & Larose, 2008; Sollberger et al., 2016).

## 2 SEIS Data Acquisition Flow

The two seismometers in the SEIS package (VBB and SP) nominally cover a combined seismic bandwidth from 0.01 to 50 Hz (Lognonné et al., 2019). Even though the instruments would be capable of measuring data at higher frequencies than 50 Hz, this upper limit is imposed by the maximum sampling rate of the acquisition hardware (100 sps). The two seismometers record continuously and the data are stored inside a buffer on‐board the lander. From there, the data are first uplinked to the relay satellites orbiting Mars (usually about two uplink passes per day) and subsequently downlinked to Earth. Due to the limited storage space of the buffer (64 Gigabit of flash storage) and data transfer bandwidth limitations, the data volume that can be transferred to Earth is restricted. The continuous seismic data is therefore down‐sampled directly on‐board the lander to a lower sampling rate before it is sent to Earth. Based on the continuous low‐rate data, event data at a higher sampling rate (up to 100 sps) can be requested for periods of time where seismic signals are observed. In this section, we describe how the data decimation process is implemented inside the space craft electronics and illustrate the changes that were implemented for the ${HP}^{3}$ hammering experiment to recover the high‐frequency information of the hammering signals.

**Figure 3:**
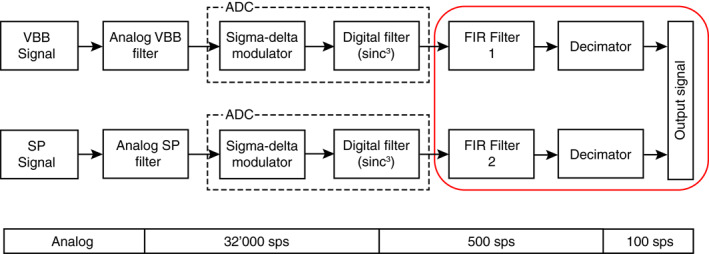
Acquisition and digitization of seismic signals recorded by the two SEIS seismometers. The filtering step in the red box can be changed from Earth by uploading different filter coefficients to the lander. Different filters can be uploaded for very broadband (VBB) and short‐period (SP) (finite‐impulse response (FIR)1 and FIR2, respectively).

The SEIS signals pass through the data acquisition and decimation flow illustrated in Figure 3. The analog voltage signal from the seismometers first passes through an analog anti‐aliasing filter, before it is digitized by the sigma‐delta analog to digital converter (ADC) on‐board the lander at a sampling rate of 32 kHz. Subsequently, the signal is passed through an additional digital ${\left(\sin\left(x\right)/x\right)}^{3}$ (also called ${sinc}^{3}$) low‐pass filter with a cut‐off frequency of 500 Hz and decimated to a sampling rate of 500 sps. The 500 sps signal is then passed through a digital FIR filter (FIR1 and FIR2 in Figure 3). Nominally, this filter is set to be a low‐pass with a cut‐off of 39.8 Hz (−3 dB half‐power point) in order to avoid aliasing in the final 100 sps data product. The FIR filters of each of the two seismometers can be individually changed by uploading new filter coefficients to the lander. During ${HP}^{3}$ hammering, we replaced the nominal FIR anti‐alias filter on the SP sensor by an all‐pass filter (FIR1 in Figure 3) in order to avoid losing the information above 50 Hz. As a consequence, the decimated signal at 100 sps contains signal up to 500 Hz, which are aliased and thus overlapping the 0–50 Hz range correspondingly.

**Figure 4:**
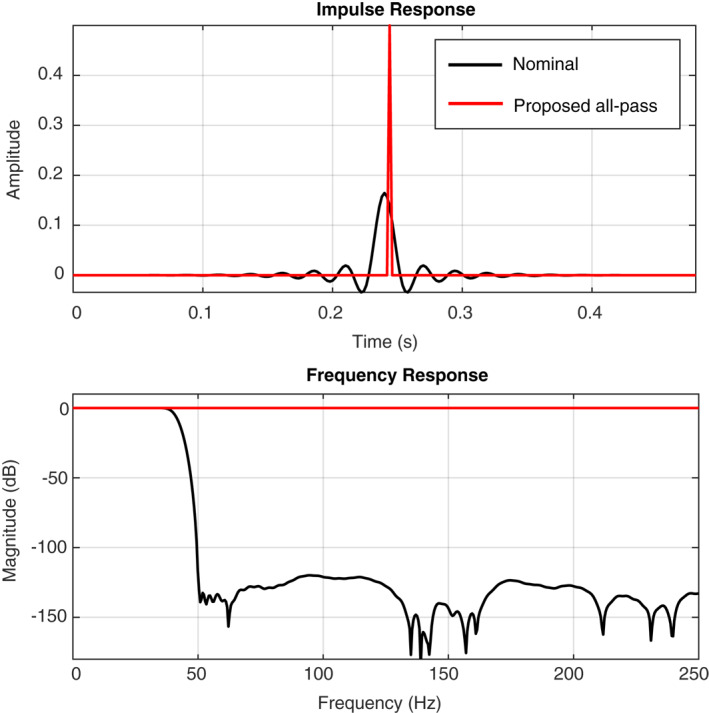
Impulse responses and frequency responses of the digital finite‐impulse response (FIR) filters implemented in the SEIS acquisition electronics.

The impulse time and frequency responses of both the nominal (39.8 Hz cut‐off) and the proposed all‐pass filters are shown in Figure 4. Note that the proposed all‐pass filter has a flat frequency response over the full bandwidth. Consequently, its impulse response in time corresponds to a single spike. Because the FIR filter coefficients are implemented in the SEIS electronics as signed 32‐bit integer numbers, the maximum possible amplitude of the spike is $\left(2^{31}-1\right)/\left(2^{32}\right)\approx 0.5$. As a consequence, the raw data need to be multiplied with a factor of 2 during the conversion from digital counts to volt, which results in the loss of 1 bit of resolution (the nominal resolution is 24 bits). Furthermore, the all‐pass filter was implemented with a group delay of 0.244 s, whereas the nominal FIR filter has a group delay of 0.24 s (see delay between the black and the red curves in the top of Figure 4).

## 3 Theory

The rules dictating the sampling of signals are governed by the Nyquist–Shannon sampling theorem (Shannon, 1948), stating that in order to reconstruct a signal from its samples, the signal must contain no information at and above the Nyquist frequency corresponding to half the sampling frequency. However, the Nyquist–Shannon sampling theorem assumes sampling of a single quantity of the underlying signal. If multiple data types, corresponding to data filtered before sampling with linearly independent filters in the domain of sampling are available, then the Nyquist–Shannon sampling criterion is relaxed proportionally to the new degrees of freedom added to solve the problem. This so‐called generalized sampling theorem (Papoulis, 1977) provides the mathematical framework for the reconstruction algorithm we propose in this paper.

In case of the ${HP}^{3}$ hammering signals, we have access to multiple realizations of approximately the same signal from subsequent mole strokes. Because the source triggering and sampling process are unsynchronized, the different realizations will appear as if they have been filtered in time with different Fourier shift filters (i.e., the 100 sps sampling comb is randomly shifted in time for each hammering signal). While the use of the generalized sampling theorem as described above relies on multiple realizations of the same signal, we allow for the reconstruction of smoothly varying signals by exploiting the inherent linear data structure when the hammer recordings are rearranged into a 2D signal (with time relative to the hammer stroke on one axis and space on the other), causing the signal to have a sparse representation in the Radon transform domain.

Reconstruction problems are inherently underdetermined (i.e., the number of samples that are sought to be recovered is always greater than the number of data points that are available to constrain the problem). Such problems thus need to be regularized in some way, which means that a priori information about the signal must be included to achieve a successful reconstruction. Recent advances in signal processing make use of signal sparsity as a priori knowledge to regularize the underdetermined reconstruction problem (Candès et al., 2006a, 2006b; Donoho, 2006). Sparsity is thereby usually described either by the $\ell_{0}$‐ or the $\ell_{1}$‐norm of the signal and penalties are given to reconstructions with high $\ell_{0}$‐ or $\ell_{1}$‐norm. A prerequisite is that the signal has a sparse representation in some transform domain and the success highly depends on the compressibility of the signal and thus the selection of the sparsifying transform (i.e., an operator mapping the signal data vector to a sparse vector). The concept of sparsity‐constrained reconstruction has been successfully applied, for example, to accelerate magnetic resonance imaging (Lustig et al., 2007) or to interpolate seismic data (Herrmann & Hennenfent, 2008).

Here, we devise a signal reconstruction algorithm using sparsity constraints. The key characteristics of the ${HP}^{3}$ seismic signals that are exploited for reconstruction are:

1. The hammering signal is highly repeatable and only slowly varying in space (depth) due to the slow penetration rate of the mole.
2. The signal sample times of repeated hammering signals are different since the trigger time of the hammer mechanism is unsynchronized with the sampling process of SEIS.

As we will demonstrate in the following, these characteristics have the effect that the hammering signals are highly compressible using a modified Radon transform. This property, in addition with the quasi‐random sub‐sampling of the signal due to the unsynchronized timing between the hammer strokes and the recording system provides the foundation for successful sparse reconstruction.

### 3.1 Signal Compressibility

Let d(t,x) be a 2D signal (e.g., seismic data) of time variable t and space variable x. The linear Radon transform allows for representing the signal as a superposition of integrals over straight lines (Radon, 1917). Each point in the transform domain (in the following referred to as τ‐p‐plane) then corresponds to the line integral of d(t,x) over the straight line with intercept time τ and slope (or slowness) p. Here, we begin with the inverse Radon transform (i.e., the operation corresponding to the summation of all points passing through a line). It can be formulated in the following way

(1) $$d\left(t,x\right)=R^{*}m_{\delta }\left(\tau ,p\right)=\int_{-\infty }^{\infty }\int_{-\infty }^{\infty }m_{\delta }\left(\tau ,p\right)\delta \left(t-\tau -px\right)d\tau dp,$$

where $m_{\delta }\left(\tau ,p\right)$ is the representation of the signal in the τ‐p‐plane, $R^{*}$ is the inverse Radon transform operator, and δ(t−τ−px) is the basis function of the transform describing lines of slope p and intercept time τ.

If the signal d(t,x) shows an underlying 2D linear structure, it will focus at sparse locations in the Radon transform domain, since the transform compresses each line to a point (i.e., the Radon transform is a sparsifying transform for such a signal).

In the following, we assume that d(t,x) is a seismic signal. Seismic data are always band‐limited due to the blurring effect caused by the band‐limited source wavelet. This reduces the temporal focusing capabilities and thus the sparsifying potential of the conventional Radon transform for seismic data. A sparser τ‐p‐representation of the data can be found when information on the seismic wavelet (i.e., the source‐time function of the seismic source) is included into the basis function of the transform (Gholami, 2017).

Let w(t) be a suitably defined, known wavelet that is, a reasonable approximation to the actual source time function of the seismic source. We now modify the basis function of the Radon transform to find a sparser τ‐p‐plane representation of the signal by including information on the wavelet (Gholami, 2017). The modified basis function now reads:

(2) w(t−τ−px)=w(t)∗δ(t−τ−px).

This new basis function is still constant along all lines of slope p but at a fixed point in space x, it is a wavelet shifted in time. This allows for a particularly good representation of seismic signals as a super‐position of band‐limited transient plane waves. It can be shown that this modified Radon transform can simply be expressed by the conventional Radon transform and an additional deconvolution with the wavelet w(t). For the inverse of this modified Radon transform, it follows that (Gholami, 2017):

(3) $$\begin{array}{cc} d\left(t,x\right) & =\;\int_{-\infty }^{\infty }\int_{-\infty }^{\infty }m_{w}\left(\tau ,p\right)w\left(t-\tau -px\right)d\tau dp\\ \; & =\;\int_{-\infty }^{\infty }\int_{-\infty }^{\infty }m_{w}\left(\tau ,p\right)\left[w\left(t\right)*\delta \left(t-\tau -px\right)\right]d\tau dp\\ \; & =\;w\left(t\right)*\int_{-\infty }^{\infty }\int_{-\infty }^{\infty }m_{w}\left(\tau ,p\right)\delta \left(t-\tau -px\right)d\tau dp=w\left(t\right)*R^{*}m_{w}\left(\tau ,p\right), \end{array}$$

where $m_{w}\left(\tau ,p\right)$ are the τ‐p‐coefficients of the signal in the modified Radon transform domain. Equation 3 makes the implementation straightforward as it allows one to use existing Radon transform routines. In the following, we make use of a recently published, fast implementation of the Radon transform (Andersson & Robertsson, 2019).

#### 3.1.1 Discrete Implementation

For the discrete implementation of this modified Radon transform, let $d\in R^{M}$, and $m\in R^{N}$ be vectors containing discrete samples of the signal coefficients in the t‐x‐ and τ‐p‐planes, respectively. The number of discrete samples are given by $M=n_{t}n_{x}$, and $N=n_{\tau }n_{p}$, with $n_{t}$ being the number of time samples, $n_{x}$ the number of samples in space, $n_{\tau }$ the number intercept times, and $n_{p}$ the number of slowness values. In the following ${\left\|.\right\|}_{p}$ is the $\ell_{p}$‐norm of a vector and (using the example of m) is defined as ${\left\|m\right\|}_{p}:={\left({\sum_{i=1}^{M}\left|m_{i}\right|}^{p}\right)}^{\frac{1}{p}}$. The discrete forward Radon transform can now be formulated in the form of an optimization problem based on Equation 3 to find the best‐fitting (in a least squares sense) τ‐p‐representation $\hat{m}$ of the signal as:

(4) $$\hat{m}=\underset{m}{\text{arg}\;\text{min}}{\left\|d-WR^{*}m\right\|}_{2}.$$

Here, $W\in R^{M\times M}$ is a block‐diagonal matrix with $n_{x}$ blocks, each block corresponding to a Toeplitz matrix $T\in R^{nt\times nt}$ that is, constructed from the wavelet by cyclic permutation. Left multiplication with W corresponds to a convolution with the wavelet. The matrix $R\in R^{M\times N}$ is the Radon transform matrix, which is readily implemented in the frequency domain with the elements given by $R_{jk}=e^{i\omega p_{j}x_{k}}$, where ω is the angular frequency. The asterisk marks the Hermitian conjugate operator. The solution of the optimization problem typically requires some form of stabilization, such as Tikhonov regularization.

**Figure 5:**
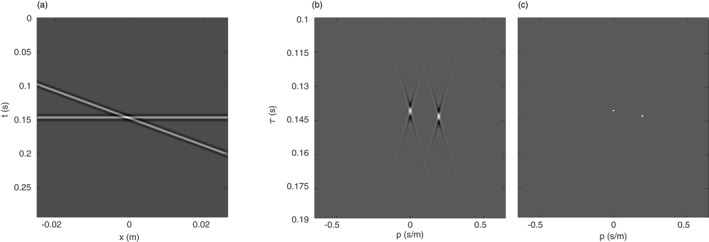
(a) Two linear band‐limited events in the space‐time domain. (b) Conventional linear Radon transform of the data in (a). (c) Sparse, modified Radon transform.

The improvements in signal compressibility that can be achieved using the modified, sparse Radon transform compared to the conventional Radon transform are illustrated in Figure 5. A synthetic signal is shown that comprises two band‐limited plane waves, the first with slowness $p_{1}$ = 0 s/m and intercept time $\tau_{1}$ = 0.15 s and the second with slowness $p_{2}$ = 0.25 s/m and intercept time $\tau_{2}$ = 0.16 s (Figure 5a). The conventional, linear Radon transform focusses the two waves at the expected locations in the τ‐p‐plane (Figure 5b). Note that the temporal resolution is limited due to the sub‐optimal choice of the basis function. Additionally, the energy of the two events smears out due to the limited aperture of the data in the space direction. The modified Radon transform accounts for the band‐limited nature of the data and allows to effectively compress each plane wave to a single point in τ‐p‐space (Figure 5c).

### 3.2 Signal Reconstruction

The reconstruction problem can be understood as a modified version of the forward Radon transform with an additional sparsity constraint. Instead of having access to the fully sampled data d, we only have access to the sub‐sampled data $b\in R^{P}$, where P is the number of sub‐samples (P≪M). For the specific problem of reconstructing the ${HP}^{3}$ seismic signals, b is under‐sampled in time and thus shows pronounced aliasing. In order to reconstruct d, we need to solve an underdetermined optimization problem. Here, we formulate the signal reconstruction problem in the form of the following basis pursuit denoise problem (BPDN), seeking for the sparsest set of τ‐p‐coefficients that explains the data with a misfit smaller than σ (an estimate of the noise level in the data) by $\ell_{1}$‐norm minimization:

(5) $$\hat{m}=\underset{m}{\text{arg}\;\text{min}}{\left\|m\right\|}_{1}s.t.{\left\|b-GWR^{*}m\right\|}_{2}\leq \sigma .$$

Here, the matrix $G\in R^{P\times M}$ is the sampling operator selecting those samples from the model that are contained in the observed data b. G can be easily constructed from the identity matrix by deleting rows corresponding to samples that are not included in b. We use the solver SPGℓ1 (van den Berg & Friedlander, 2009, 2011), which allows for an efficient solution of the BDPN problem by breaking it down into a series of so‐called LASSO problems, each of the form

(6) $$\hat{m}=\underset{m}{\text{arg}\;\text{min}}{\left\|b-GWR^{*}m\right\|}_{2}s.t.{\left\|m\right\|}_{1}\leq \rho_{k},$$

where $\rho_{k}$ is the $\ell_{1}$‐norm constraint for the solution of the $k^{th}$ LASSO problem (k being the iteration counter). For a well‐defined series of constraints $\rho_{0}<\rho_{1}<\ldots <\rho_{k}$, the solution converges to the solution of the BDPN problem (Equation 5), as soon as the least‐squares misfit reaches the pre‐defined error level σ. It turns out that the series of $\ell_{1}$‐norm constraints $\rho_{k}$ can be readily defined using a Newton root‐finding method on the Pareto curve (van den Berg & Friedlander, 2009). The Pareto curve traces the optimal trade‐off between the least‐squares misfit and the $\ell_{1}$‐norm of the solution. It is convex, decreasing and continuously differentiable. Each solution of the k LASSO problems lies on the Pareto curve and the slope of the curve at that point can be expressed in closed form (van den Berg & Friedlander, 2009). This property is used to find the optimal $\ell_{1}$‐constraint for the next LASSO problem using Newton's method. At each iteration, the new LASSO problem can be “warm‐started” using the solution of the previous iteration. For details on this procedure, we refer the reader to (van den Berg & Friedlander, 2009, 2011) and Appendix B in (Lin & Herrmann, 2013). After convergence, the reconstructed signal $\hat{d}$ can be found by $\hat{d}=WR^{*}\hat{m}$.

Algorithm 1 **Reconstruction of ${HP}^{3}$ hammering seismic signals**

1

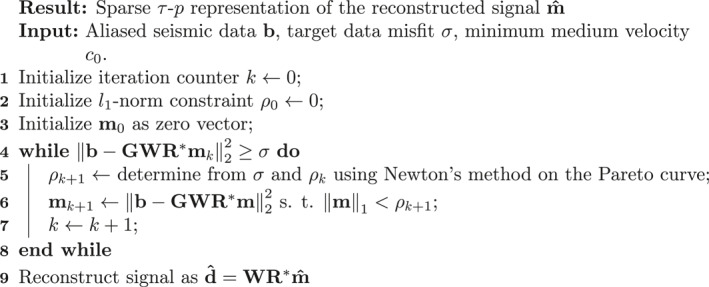

There are three user‐specified input parameters for the reconstruction algorithm: (a) the target data misfit σ (i.e., the noise level in the data), which can be estimated directly from the data during periods where the hammer is not active, (b) the source wavelet, and (c) the slowness range that is, used to parameterize the Radon transform. This slowness range is naturally bounded by the lowest seismic velocities in the medium, which are typically shallow S‐wave velocities. In the τ‐p plane, all signal must thus be contained in the cone‐shaped, convex set $C=\left\{(\tau ,p):\left|p\right|\leq \frac{1}{c_{0}}\right\}$, where $c_{0}$ is the lowest seismic velocity in the medium. This puts an additional constraint on the reconstructed signal (i.e., it must only have support within C). Everything outside the set C corresponds to noise. On Mars, the shallow near‐surface seismic shear wave velocities are expected to be very low, on the order of $c_{0}=$ 40–50 m/s (Morgan et al, 2018). The proposed reconstruction algorithm is summarized in Algorithm 1.

## 4 Numerical Example

**Figure 6:**
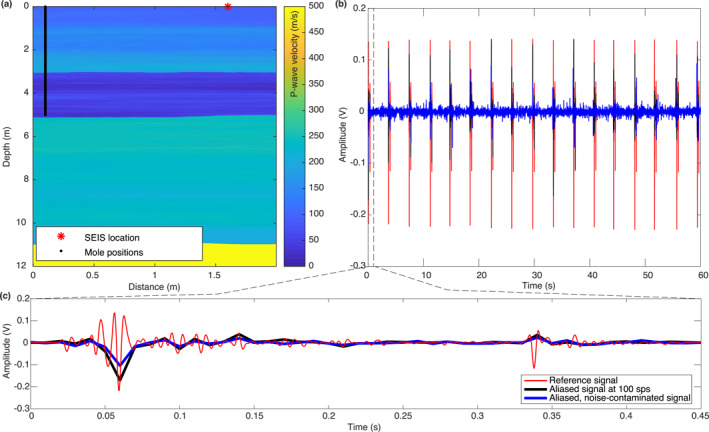
(a) Input velocity model for synthetic data computation. (b) Synthetic data set emulating 60 s of ${HP}^{3}$ hammering recorded by SEIS. (c) Zoom‐in into the first hammer stroke.

In order to illustrate the reconstruction algorithm, we generated synthetic data using a time‐domain finite‐difference method for heterogeneous elastic media (Virieux, 1986). We used a near‐surface velocity model that is, based on mechanical tests conducted on regolith simulants in the laboratory (Delage et al., 2017; Morgan et al., 2018). Additionally, we added 2D stochastic velocity fluctuations based on a Von Kármán model (Goff & Holliger, 2003; Korn, 1993) in order to simulate a heterogeneous subsurface. For illustration, the P‐wave velocity distribution of the final model is given in Figure 6.

Making use of source‐receiver reciprocity, we then generated synthetic seismic data for a total of 1,000 mole positions in a single computation by placing a vertically directed force source at the location of SEIS (marked by a red asterisk in Figure 6a) and 1,000 receivers spaced vertically at 5 mm from the surface down to a depth of 5 m at a lateral offset from SEIS of 1.5 m at the surface (receivers marked by the black line in Figure 6a). We used an experimentally determined source‐time function from an analog experiment on Earth with a dominant frequency of about 150 Hz. We then interpolated the computed data to a receiver spacing of 1 mm in order to emulate the actual penetration rate of the mole. Finally, we concatenated all of the resulting 5,000 hammering signals to a single, continuous record. The time differences between individual hammer strokes were chosen from a normal distribution with a mean value of 3.7 s and a standard deviation of 0.1 s to mimic the real duration and variations of the mole's hammering cycle (Spohn et al., 2018).

The first 60 s of the resulting record are shown in Figure 6b. A zoom‐in showing the first hammer stroke is provided in Figure 6c. The red line marks the unaliased data sampled at 32 kHz before it would pass through the down‐sampling flow on‐board the lander (see Figure 3). The black line marks the same signal after passing through the on‐board acquisition flow using the proposed all‐pass FIR filter in the final step (see Figure 4) before decimating the signal to 100 sps. Note that the signal is now severely undersampled (aliased). We then additionally added noise to the signal giving the signal marked by the blue line in Figures 6b and 6c, which now corresponds the final input that we used to test the proposed reconstruction algorithm. The added noise corresponds to actual noise that was measured on Mars during an early phase of the InSight mission with the proposed all‐pass FIR filter on the SP sensor.

**Figure 7:**
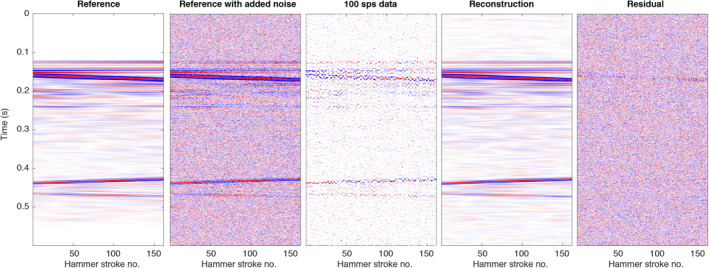
Application of the proposed reconstruction algorithm to a synthetic test data set (see text for details).

For reconstruction, we then sorted the data into a 2D matrix, where each column corresponds to a single hammer stroke signal (Figure 7). Note that the zero‐time corresponds to the time when the hammer strike occurs. This zero‐time time can be retrieved with an accuracy of 1.7e−3 s from the measurements of an accelerometer that is, mounted inside the mole (Spohn et al., 2018). The left panel in Figure 7 shows the assembled data matrix of the unaliased reference signal at a sampling rate of 2,000 sps. For the test, we only use 10 min of data (160 hammer strokes). Note that the signal is only slowly varying with depth (due to the slow penetration rate of the mole and the repeatability of the hammering signal), resulting in the linear structure that is, exploited by the proposed reconstruction algorithm.

The second panel from the left shows the reference data with added real noise as measured on Mars with the all‐pass FIR filter. The samples contained in the 100 sps, aliased data (input to the reconstruction algorithm) are given in the third panel. Note that even though the signal is regularly sampled in time (at 100 sps), the sampling in 2D appears to be close to random. This is due to the fact that the timing of the hammer strokes is not synchronized with the SEIS recording system. The respective sub‐sampling of each hammer signal depends on the duration of the hammer cycle (subject the small variations caused by ambient conditions) and the relative positions of the mole and SEIS. As a result, each repeated signal is sub‐sampled differently, resulting in the random 2D sampling pattern, which provides an optimal basis for the proposed reconstruction algorithm using the generalized sampling theorem (Papoulis, 1977).

We then estimated an average source‐time function from the aliased data, by combining the samples of 20 neighboring hammer stroke signals to a single trace at 2,000 sps, from which we extracted a wavelet by time‐windowing the first‐arrival.

The output of the proposed reconstruction algorithm (reconstructed to a sampling rate of 2,000 sps) is shown in the fourth panel in Figure 7. For the parametrization of the Radon transform, we used slowness values ranging from −0.04 s/m to +0.04 s/m (reconstruction is limited to events with a minimum absolute apparent velocity greater than 25 m/s). The high‐frequency signal is accurately retrieved by the reconstruction algorithm. Note that random noise appears to be suppressed in the output compared to the noise‐contaminated input data. This is a positive side‐effect of the proposed reconstruction approach owing to the properties of the Radon transform. The integration along straight lines will cause coherent energy (signal) to add constructively and focus in the Radon domain, while random noise tends to spread out over the whole domain and cancel out. By promoting sparsity of the signal in the Radon domain, the signal is effectively denoised since only the largest coefficients (corresponding to signal) are kept in the reconstruction. The rightmost panel in Figure 7 shows the reconstruction residual (i.e., the difference between the reference and the reconstructed signal). Note that the residual is mainly dominated by noise, indicating that the underlying signal was successfully reconstructed. Some minor reconstruction errors seem to be present at the edges for the events with the lowest apparent velocity. These errors are likely Radon transform artifacts (linear flares) caused by the truncation of the data set (Andersson & Robertsson, 2019).

**Figure 8:**
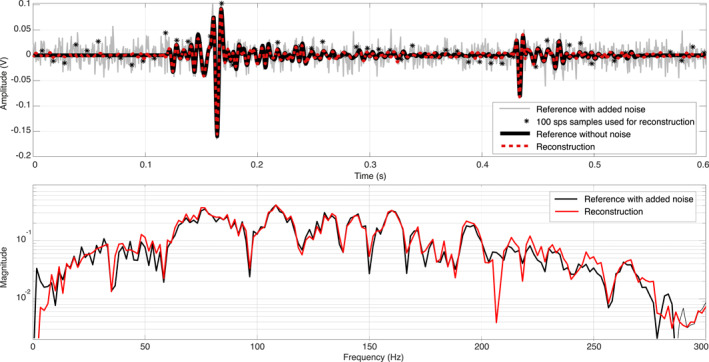
Reconstruction result illustrated on a single hammering signal (hammer stroke no. 60 in Figure 7). Top: Time domain result. Bottom: Frequency domain result.

In order to further illustrate the performance of the reconstruction algorithm, we provide the result for a single hammering signal in Figure 8. The top panel shows the result in the time domain. The black line corresponds to the fully sampled reference signal. The noise‐contaminated samples at 100 sps that are used for the reconstruction are marked by asterisks. The reconstructed signal is plotted in red. Note that the reconstruction result is close to the noise‐free reference signal (black). An inspection of the amplitude spectrum (bottom panel) confirms that the reconstruction appears to recover the underlying signal throughout the entire signal bandwidth.

### 4.1 Sensitivity on the Source Wavelet

The task of directly estimating the source wavelet from the data can be challenging in cases where the signal is dominated by strong resonances that lead to a quasi‐monochromatic appearance of the data. Additionally, in certain cases the waveform of first‐arriving wave does not accurately represent the source‐time function (e.g. due to interference of different arrivals). It is thus critical to evaluate how much the reconstruction results suffer from a poorly estimated wavelet. To address this issue, we performed a sensitivity analysis using the synthetic data set described above (noise‐free version).

**Figure 9:**
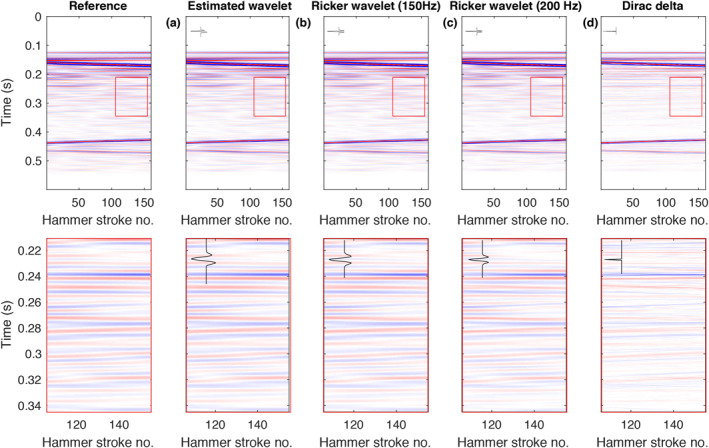
Impact of the user‐prescribed source wavelet on the reconstruction result. Top row: Full data set. Bottom row: Zoom‐in into the area labeled with a red box. The wavelet used for reconstruction is shown in the top left corner. The shown reconstruction results are obtained using (a) a wavelet estimated directly from the aliased data (see text for details), (b) a Ricker wavelet with the correct center frequency of the signal of 150 Hz, (c) a Ricker wavelet with an over‐estimated center‐frequency of 200 Hz, and (d) a Dirac delta function.

Reconstruction results are shown in Figure 9 in comparison to the reference for different strategies of choosing the wavelet basis: (a) the wavelet is directly estimated from the aliased data by combining samples from neighboring traces as described above, (b) the wavelet is pre‐described by a Ricker wavelet with a center‐frequency corresponding to the actual dominant frequency in the data (150 Hz), (c) the wavelet is pre‐described by a Ricker wavelet with an overestimated center‐frequency (200 Hz), and (d) the wavelet is simply set to a Dirac delta function. The first three approaches (a)–(c) all yield almost identical results with a residual reconstruction error smaller than 1 percent compared to the ground truth. Thus, a slight error in the estimation of the wavelet only has a minor impact on the reconstruction results. Choosing a Dirac delta function as wavelet basis clearly leads to poorer results (reconstruction error of about 10%). Nevertheless, a more suitable wavelet can easily be found from such an initial result by Wiener deconvolution, as proposed by Gholami (2017). The wavelet can be iteratively adapted until no change in the reconstruction result is observed.

The relatively minor impact of the wavelet on the reconstruction quality can be explained by the way the data is compressed by the Radon transform. The major contribution to the compression comes from the mapping of near‐horizontal (slowly varying) features in the horizontal (spatial) direction to points in the Radon transform domain. In comparison, the compression of features in the temporal direction due to the choice of the wavelet basis only amounts to a minor contribution of the overall compression rate.

## 5 Mars Data Example

We applied the proposed reconstruction algorithm to actual signals recorded on Mars (InSight Mars SEIS Data Service, 2019). The ${HP}^{3}$ mole began its hammering operations on Mars on February 28, 2019. After about the first five minutes of hammering (≈80 strokes), the mole got stuck at a depth of about 30 cm and did not make any significant progress in depth anymore. In an attempt to recover the mole and to extract diagnostics on the cause of the encountered anomaly, the mole has conducted close to 10,000 hammer strokes that did not result in any significant progress in depth. All strokes were recorded by both SEIS seismometers with a high signal‐to‐noise ratio.

We apply our reconstruction algorithm to data from a short hammering session, consisting of 200 hammer strokes (about 12 min of hammering) carried out on Mars on March 26, 2019. During this hammering session, the SP sensor was operated using the proposed all‐pass FIR filter (Figure 4) while the VBB sensor was operated with the nominal anti‐aliasing filter (no signal above 50 Hz recorded). Due to the encountered problems, the mole did not make any noticeable progress in depth during the 200 hammer strokes.

**Figure 10:**
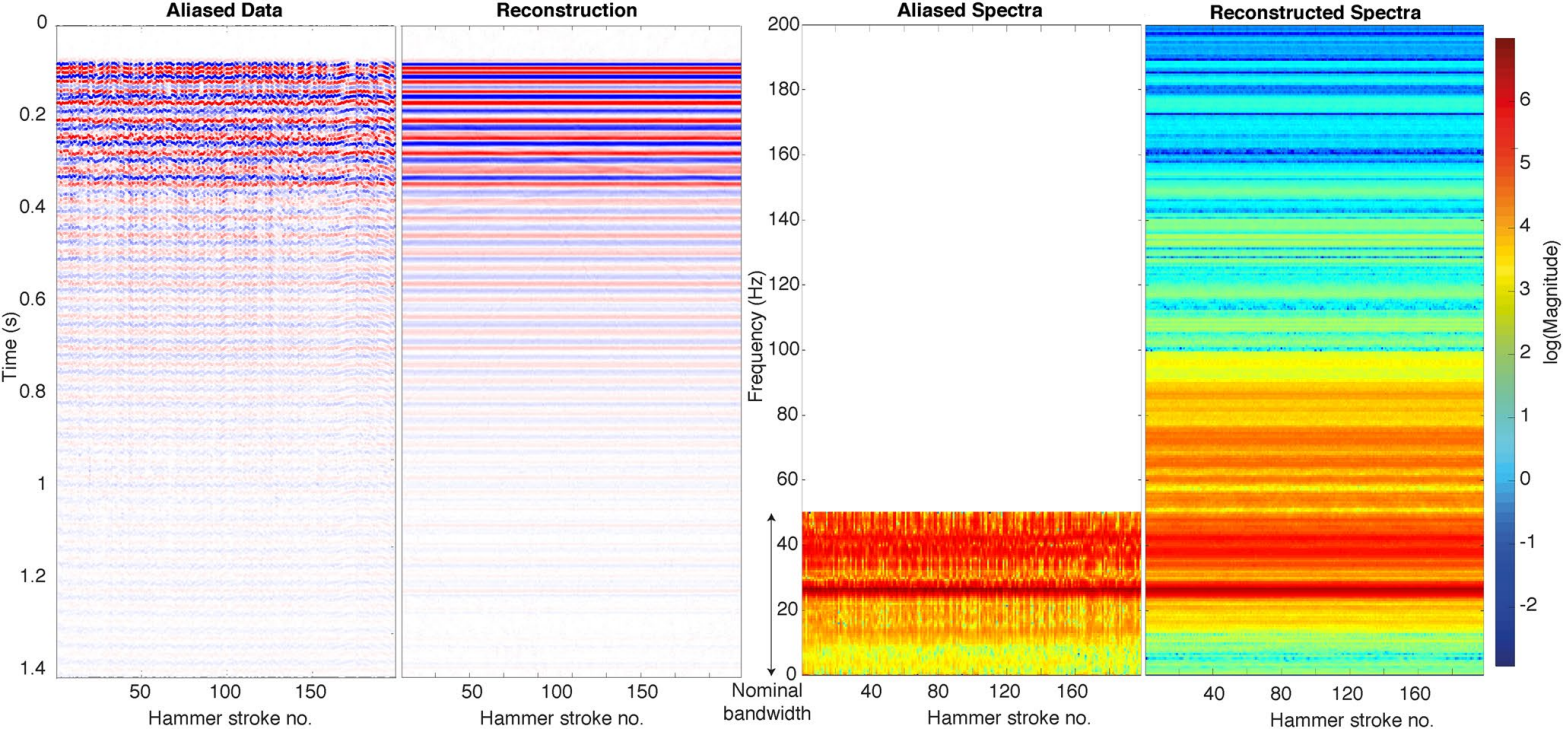
Application of the proposed signal reconstruction algorithm to actual data recorded on Mars. Left two panels: Time domain result. Right: Frequency domain result.

The data are characterized by a high signal‐to‐noise ratio on both the SP and VBB sensor. The VBB data confirmed that the hammering signal is highly repeatable. The aliased, 100 sps signals (recorded on the SP sensor) of all 200 strokes arranged in a 2D matrix are shown in the left panel in Figure 10. Since the accelerometers mounted inside the mole need to be calibrated and did not provide sufficiently precise information on the trigger time of the hammer strokes for the first few hammer sessions, we had to rely on a different approach to align the data: We first upsampled the 0–50 Hz data from the VBB sensor to 2,000 sps and then used a cross‐correlation procedure to align the individual hammer stroke signals. This procedure allowed us to find the relative shifts of the 100 sps subsampling comb function from stroke to stroke, which we used to determine the subsampling operator (matrix G in Equation 5). Note that, as a result of this procedure, the zero‐time in Figure 10 does not correspond to the actual hammering time. For later sessions, we could directly use the calibrated trigger time from the mole.

The reconstruction result for the 200 hammer strokes is given in the second panel in Figure 10. As expected, the signal characteristics do not significantly change between different hammer strokes. Differences in the signal at later times (later than 0.15 s) are likely caused by variations in the timing of the second and third sub‐stroke of the hammering mechanism (Spohn et al., 2018). The frequency spectra displayed in the right‐most panel of Figure 10 illustrate that the signal contains a significant amount of information above the original Nyquist frequency of 50 Hz. Note that this information would have been lost using the nominal anti‐aliasing filters. The low‐frequency portion of the signal is dominated by long‐lasting reverberations following the first arrival. These reverberations have a dominant frequency of about 25 Hz, as can be seen from the frequency spectra in the right panel of Figure 10 (distinct peak at 25 Hz for each stroke). The successful reconstruction of these reverberation illustrate that also quasi‐monofrequent signals can be recovered well by our algorithm. The cause of the reverberations is currently under investigation.

### 5.1 Results on Martian Near‐Surface Properties

The high‐sampling rate data that we obtained by applying the proposed reconstruction algorithm allowed us to successfully estimate the P‐wave velocity in the top first meter of the Martian regolith. The travel time of the first‐arriving wave was determined to be 9.40±2.68 milliseconds over a distance of 1.11 m (with the mole tip at a depth of 33 cm pointing toward SEIS) resulting in a P‐wave velocity of $118\pm 34\;{ms}^{-1}$ (Lognonné et al., 2020). Note that the extracted travel time is shorter than the nominal SEIS sampling interval of 10 milliseconds, which illustrates the importance of the proposed reconstruction algorithm for the seismic analysis of the mole hammering data.

Unfortunately, the encountered anomaly with the mole could not be resolved and mole operations had to be terminated in January 2021 with the mole remaining stuck at a depth of about 40 cm. Unexpected soil properties were identified to be the cause of the anomaly providing insufficient friction to compensate for recoil during hammering (Hudson et al., 2020). As a result, the high‐resolution active seismic data are limited to hammer strokes that all occurred at approximately the same depth. This exacerbates the extraction of geological information from the data beyond the estimation of P‐wave velocities. The additional identification and imaging of reflections from subsurface discontinuities would require the observation of seismic signals originating from various mole depths. Nevertheless, our results constitute the first in‐situ measurement of regolith seismic velocities on Mars. Limited telemetry bandwidth will remain an issue for the foreseeable future in space exploration and solutions to reduce the data volumes, such as the sampling and reconstruction approach described in this paper, could therefore prove valuable for future space missions.

## 6 Conclusion

The high‐frequency information of the ${HP}^{3}$ hammering signal (frequencies above the nominal Nyquist frequency of 50 Hz) can be accurately recovered by the proposed reconstruction algorithm. Since the hammering time of the mole is uncorrelated with the sampling time of the seismometer, multiple realizations of approximately the same signal are recorded, where each realization appears to be filtered with a Fourier‐shift filter. This allows for the recovery of the full‐bandwidth signal by the application of the generalized sampling theorem. Since the signal is smoothly varying with depth as the mole slowly penetrates into the subsurface, we additionally make use of the Radon transform, which allows us to account for the resulting slope in the 2D signal. The maximum rate of change of the signal with depth is prescribed by the lowest propagation velocities in the Martian ground, defining a limited area in the Radon transform domain where the signal has support. Reconstruction is then achieved by finding the sparsest set of Radon coefficients in this area that fit the data within the noise, allowing us to unwrap several orders of aliasing. We have demonstrated that this approach is robust also in the presence of high levels of random noise due to the inherent properties of the Radon transform.

The proposed reconstruction algorithm could be adapted to similar problems of repeated and only smoothly varying aliased and (quasi‐)randomly sampled signals in situations where sufficiently dense sampling along one dimension is not possible.

## Data Availability

High‐rate seismic data from ${HP}^{3}$ hammering obtained using the reconstruction algorithm described in this paper is made available in a public repository at https://doi.org/10.5281/zenodo.4001920.
